# Pretreatment diffusion-weighted imaging for prediction of relapsed and refractory primary central nervous system lymphoma

**DOI:** 10.3389/fneur.2023.1227607

**Published:** 2023-08-10

**Authors:** Hsi-Cheng Chien, Lee-Ren Yeh, Kuo-Chuan Hung, Sher-Wei Lim, Chung-Yu Cheng, Yu-Chang Lee, Jeon-Hor Chen, Ching-Chung Ko

**Affiliations:** ^1^Department of Medical Imaging, Chi Mei Medical Center, Tainan, Taiwan; ^2^Department of Medical Imaging, E-Da Hospital, Kaohsiung, Taiwan; ^3^Department of Medical Imaging and Radiological Sciences, College of Medicine, I-Shou University, Kaohsiung, Taiwan; ^4^School of Medicine, College of Medicine, I-Shou University, Kaohsiung, Taiwan; ^5^Department of Anesthesiology, Chi Mei Medical Center, Tainan, Taiwan; ^6^Department of Hospital and Health Care Administration, College of Recreation and Health Management, Chia Nan University of Pharmacy and Science, Tainan, Taiwan; ^7^Department of Neurosurgery, Chi Mei Medical Center, Chiali, Tainan, Taiwan; ^8^Department of Nursing, Min-Hwei College of Health Care Management, Tainan, Taiwan; ^9^Department of Radiological Sciences, University of California, Irvine, Irvine, CA, United States; ^10^Department of Health and Nutrition, Chia Nan University of Pharmacy and Science, Tainan, Taiwan

**Keywords:** CNS lymphoma, relapse, refractory, ADC, DWI, MRI

## Abstract

**Objectives:**

A subset of primary central nervous system lymphoma (PCNSL) has been shown to undergo an early relapsed/refractory (R/R) period after first-line chemotherapy. This study investigated the pretreatment clinical and MRI features to predict R/R in PCNSL, emphasizing the apparent diffusion coefficient (ADC) values in diffusion-weighted imaging (DWI).

**Methods:**

This retrospective study investigated the pretreatment MRI features for predicting R/R in PCNSL. Only patients who had undergone complete preoperative and postoperative MRI follow-up studies were included. From January 2006 to December 2021, 52 patients from two medical institutions with a diagnosis of PCNSL were included (median follow-up time, 26.3 months). Among these, 24 (46.2%) had developed R/R (median time to relapse, 13 months). Cox proportional hazard regression analyses were performed to determine hazard ratios for all parameters.

**Results:**

Significant predictors of R/R in PCNSL were female sex, complete response (CR) to first-line chemotherapy, and ADC value/ratio (*p* < 0.05). Cut-off points of ADC values and ADC ratios for prediction of R/R were 0.68 × 10^−3^ mm^2^/s and 0.97, with AUCs of 0.78 and 0.77, respectively (*p* < 0.05). Multivariate Cox proportional hazards analysis showed that failure of CR to first-line chemotherapy and low ADC values (<0.68 × 10^−3^ mm^2^/s) were significant risk factors for R/R, with hazard ratios of 5.22 and 14.45, respectively (*p* < 0.05). Kaplan–Meier analysis showed that lower ADC values and ratios predicted significantly shorter progression-free survival (*p* < 0.05).

**Conclusion:**

Pretreatment ADC values in DWI offer quantitative valuable information for the treatment planning in PCNSL.

## Introduction

Primary central nervous system lymphoma (PCNSL) is a rare subtype of non-Hodgkin’s lymphoma (NHL), and accounts for 3–4% of central nervous system (CNS) tumors and 1–2% of NHLs (1, 2). PCNSL is typically confined to the brain, cerebrospinal fluid, and eyes without evidence of systemic spread. Diffuse large B- cell lymphoma (DLBCL) is the most common subtype of PCNSL (3). Currently, no optimal standard salvage regimen is available in clinical practice. High-dose (HD) methotrexate (MTX)-based chemotherapy is the cornerstone of therapy in PCNSL, although performing whole-brain radiotherapy (WBRT) after chemotherapy yields a better survival rate than chemotherapy alone (4, 5). However, in patients who have received this type of combined therapies, the incidence of radiotherapy-induced neurotoxicity is shown to increase (6). Although MTX-based chemotherapy and WBRT are considered to be effective therapies for PCNSL (4, 5), up to 50–60% of patients eventually had relapsed/refractory (R/R) disease, and the prognosis for R/R PCNSL remains poor (7, 8).

The International Extranodal Lymphoma Study Group (IELSG) proposed a prognostic scoring system comprising age, Eastern Cooperative Oncology Group performance status, serum lactate dehydrogenase (LDH) level, cerebrospinal fluid (CSF) protein concentration, and extent of the involvement of deep brain structures (9). However, the IELSG score is not always available for all PCNSL patients due to insufficient CSF study, which is necessarily invasive and is shown to be associated with complications. Magnetic resonance imaging (MRI) is a standard diagnostic modality for brain tumors, and several studies have reported that PCNSL demonstrates characteristic MRI features (10). Conventional MRI findings such as tumor size, infratentorial localization, and non-enhancing T2-fluid attenuated inversion recovery (FLAIR) hypersignal lesion have been reported as important variables associated with the prognosis of PCNSL (11). However, most findings have been presented in qualitative terms, and inter-reader variations may occur during MRI interpretation. The apparent diffusion coefficient (ADC) values generated from diffusion-weighted MR imaging (DWI) have been widely used as an imaging biomarker to evaluate the prognosis in various intracranial and extracranial tumors (12). Theoretically, DWI provides biomedical information in tissues of interest based on measurement of thermally induced water diffusion shown by quantitative ADC values (13). Therefore, unusual findings on ADC can serve as an early predictor of biological abnormality (12). After literatures review, the association between ADC values and clinical outcomes in PCNSL are only reported in rare studies with small samples, and the results are inconsistent between the existing studies (14–18). Also, no definite pretreatment ADC threshold values have yet been reported for prediction of R/R PCNSL. This study aimed to investigate the clinical and MRI features for the predicting R/R PCNSL, emphasizing the ADC values and ratios acquired by using DWI.

## Materials and methods

### Ethics statement

The protocol for the present retrospective study was approved the Institutional Review Board of our hospital (IRB number: 10902-009). The requirement for signed informed consent of subjects was waived because patient data in this retrospective study would not affect the healthcare of the included patients. All patients’ records were anonymized and de-identified before analysis.

### Patient selection

A total of 52 consecutive patients (23 men and 29 women with median age 64 years) diagnosed with PCNSL between January 2006 and December 2021 were included from two medical institutions (Chi Mei Medical Center and E-Da Hospital) based on the following criteria: histopathologic confirmation of PCNSL; with pretreatment brain MRI, including DWI and corresponding ADC imaging, and post-treatment brain MRI follow-up; no evidence of human immunodeficiency virus infection or other immunodeficiencies; no evidence of systemic involvement based on bone marrow biopsy and positron emission tomography (PET)/computed tomography (CT) scans of the chest, abdomen, and pelvis. Patients missing any of these data or having evidence of systemic involvement were excluded. All patients had a pathologic diagnosis of DLBCL as defined by the World Health Organization and had received identical MTX-based induction chemotherapy treatment. Patients’ clinical data were reviewed, including medical history, age, sex, pathological diagnosis, treatment methods, response, and survival.

### Treatments and response evaluation

First-line therapy consisted of HD-MTX (8 g/m^2^/day 1)-based regimens for all patients. Every patient received HD-MTX-based chemotherapy for 4–6 cycles (induction). In addition to HD-MTX, 25 patients also received rituximab (375 mg/m^2^/day 1) for induction. Concurrent dexamethasone was used for control of neurologic signs and symptoms. Intravenous leucovorin after MTX and granulocyte colony-stimulating factor (G-CSF) were given if necessary. For patients receiving autologous stem cell transplantation (auto-SCT) for consolidation therapy, thiotepa-based conditioning regimens were performed followed by stem-cell transplantation. WBRT (4,500 cGy) was performed after first-line chemotherapy as salvage treatment for RR disease. Patients’ responses to treatment were assessed using the International PCNSL Collaborative Group criteria, based on imaging, corticosteroid use, CSF cytology, and slit lamp examination for cases with CSF or ocular involvement (19). Of the included 52 patients, 22 patients underwent consolidation therapy with WBRT, and 2 patients received both WBRT and auto-SCT due to R/R disease.

Patients’ response to treatment was evaluated by two radiologists (H.C.C., 4 years of radiological experience; C.C.K., 12 years of neuroradiological experience) by comparing the post-treatment brain MRI findings. Both readers were blinded to the clinical data of the included patients. For equivocal cases, agreement was arrived at by consensus. For response to first-line chemotherapy, patients who achieved a complete response (CR) were defined as the disappearance of all contrast-enhancing tumors on follow-up MRI. Partial response (PR) was defined as at least 50% decrease in contrast-enhancing tumor volume (20). Progressive disease (PD) was defined as more than 25% increase in contrast-enhancing tumor volume or the development of new enhancing tumors on follow-up MRI or involving the eye or CSF. Stable disease (SD) was defined as less than a PR but is not progressive disease (14, 15, 19, 20). Brain MRI follow-up was scheduled every 3 months after treatment. Isolated CNS or systemic tumor relapse was evaluated by contrast-enhanced CT/MRI, PET, and clinical/laboratory data according to the Report of an International Workshop to standardize baseline evaluation and response criteria for PCNSL (19). CR, PR, and SD were regarded as successful treatments. Progression-free survival (PFS) was determined from the date of successful treatments until tumor relapse, disease progression, or death from any cause during follow-up or later.

### Imaging acquisition

Preoperative brain MRI images were acquired using a 1.5-T (*N* = 48) (Siemens Avanto or Siemens Aera, Siemens Corp., Malvern, PA, United States; or GE Signa, GE Healthcare, Chicago, IL, United States) or a 3-T (GE Discovery MR750) (*N* = 4) MR scanner, equipped with eight-channel head coils in each machine. All included patients received the following MRI scanning sequences: axial spin echo T1-weighted imaging (T1WI), axial fast spin-echo T2-weighted imaging (T2WI), axial T2-fluid attenuated inversion recovery (FLAIR), axial DWI and ADC map, and axial with coronal contrast-enhanced (CE) T1WI. The DWI (*b* = 1,000 or 1,500 s/mm^2^) was performed by sequential application in the x, y, and z directions, and ADC maps were obtained from these imaging data. CE images obtained in axial and coronal T1WI were performed after intravenous administration of 0.1 mmol/kg of body weight of gadobutrol (Gadovist) or gadoterate meglumine (Dotarem). The detailed MR imaging protocols are shown in Supplementary file S1.

### Image analysis and measurement of ADC values

For pretreatment MRI features, two radiologists (H.C.C. and C.C.K.) performed qualitative and quantitative imaging measurement of PCNSL on conventional and diffusion-weighted MRI. In cases of multifocal tumors, the lesion largest in size was chosen for measurement. Tumor necrosis and leptomeningeal seeding was identified on CE T1WI. Peritumoral edema was identified as the peritumoral hyperintense area on T2 FLAIR images. Intratumoral hemorrhagic change was determined by high T1WI signal and susceptibility artifacts on GRE. For measurement of ADC values, the regions of interest (ROI) with area from 20 to 64 mm^2^ (mean 34 ± 4 mm^2^) were placed in the solid enhanced tumor part and avoided volume averaging with necrosis and hemorrhage that might influence the ADC values. For normalization of individual variance, an equal ROI was placed in contralateral normal-appearing cerebral white matter (NAWM) in each patient, and ADC ratio was calculated by dividing the ADC in the tumor by the ADC in contralateral NAWM in each patient as previously described (21–23).

### Statistical analysis

Statistical analyses were performed using the statistical package SPSS (V.24.0, IBM, Chicago, Illinois, United States). The Chi-square test (or Fisher exact test) and the Mann–Whitney U test were used to evaluate categorical and continuous data, respectively. Receiver operating characteristic (ROC) and area under the curve (AUC) analyses of the ADC value and ADC ratio was performed to discriminate between patients with and without R/R. Kaplan–Meier analysis was used to assess PFS. The log-rank test was used to assess the significance in the R/R rates. Univariate and multivariate Cox proportional hazard regression analyses were performed to determine the hazard ratio of each parameter. A *p-*value < 0.05 was considered statistical significance. The interobserver reliability was determined by using the Cohen k coefficient and intraclass correlation coefficient (ICC) for categorical and continuous data, respectively. The Cohen k coefficient and ICC were evaluated using the methods described by Landis and Koch (24). Cohen k coefficient values of 0.81–0.99 were obtained for categorical MRI, indicating almost perfect reproducibility. An ICC of 0.76–0.92 was obtained for the continuous data. Due to the substantial to almost perfect reproducibility in ICC, the subsequent statistical evaluation in continuous data was performed by using the mean value calculated from both raters.

## Results

### Clinical data and MRI findings

**Figure 1:**
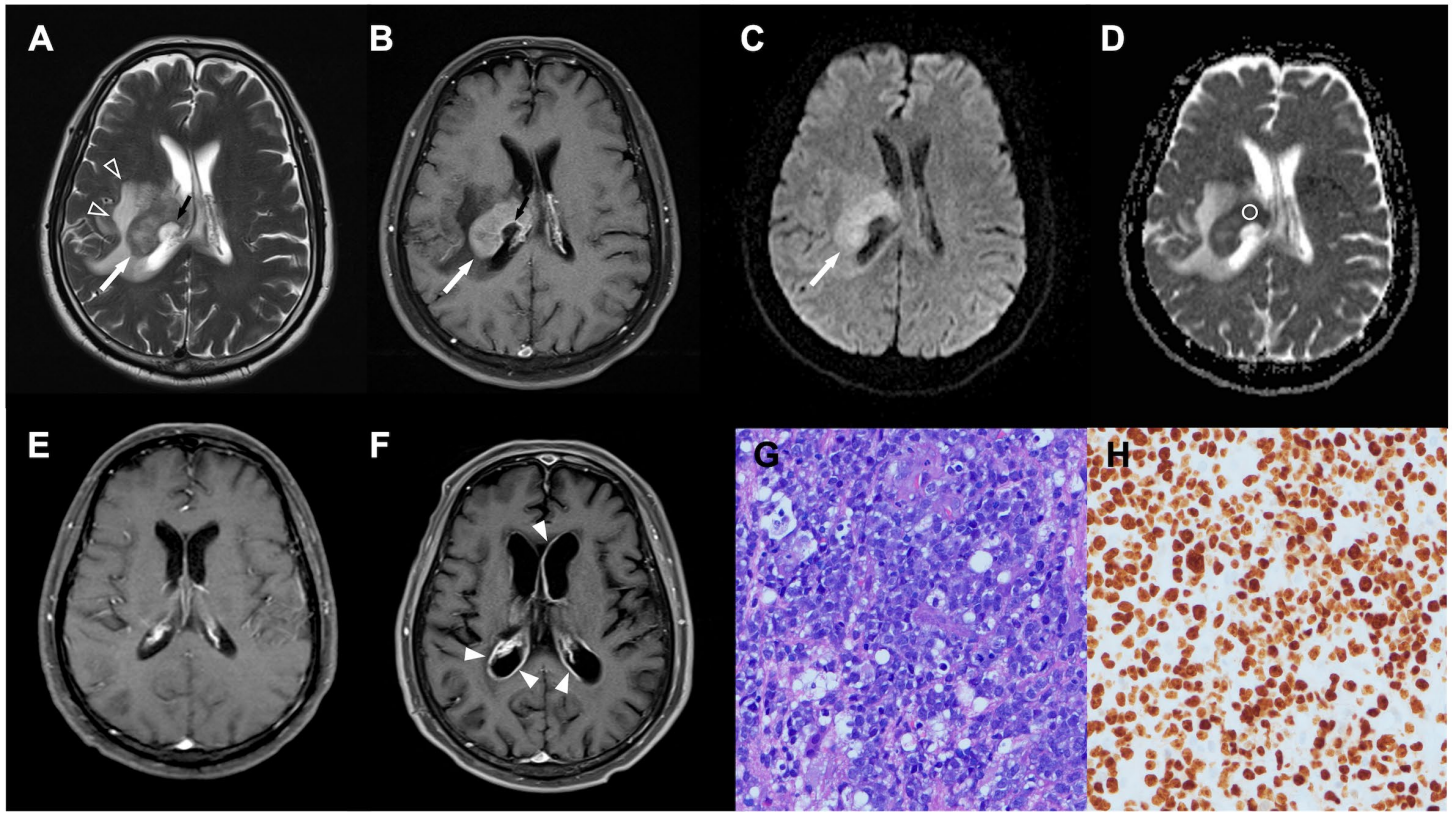
A 64-year-old man with pathologically confirmed primary central nervous system lymphoma (PCNSL). **(A,B)** Axial T2WI **(A)** and contrast-enhanced (CE) T1WI **(B)** showing an enhanced tumor (white arrow) arising from the right thalamus, with peritumoral edema (open arrowhead) and poorly enhanced central necrosis (black arrow). **(C)** Diffusion-weighted image (DWI) showing hyperintensity in the solid part of the tumor (white arrow). **(D)** Measured apparent diffusion coefficient (ADC) value (circular region of interest) and ADC ratio were 0.61 × 10 mm^2^/s and 0.86, respectively. **(E)** Complete response was achieved after first-line chemotherapy. **(F)** Recurrent tumor (arrowheads) along bilateral ventricles were observed 8 months after complete response. **(G)** Diffuse large B cell lymphoma with round nuclei, high nuclear cytoplasmic ratio, and numerous apoptotic bodies was observed in pathologic examination (HE staining, × 400). **(H)** The proliferation rate determined from the immunohistochemical Ki-67 labeling index was 90%. Only the dark brown stained nuclei were considered immunopositive (original magnification 400).

**Figure 2:**
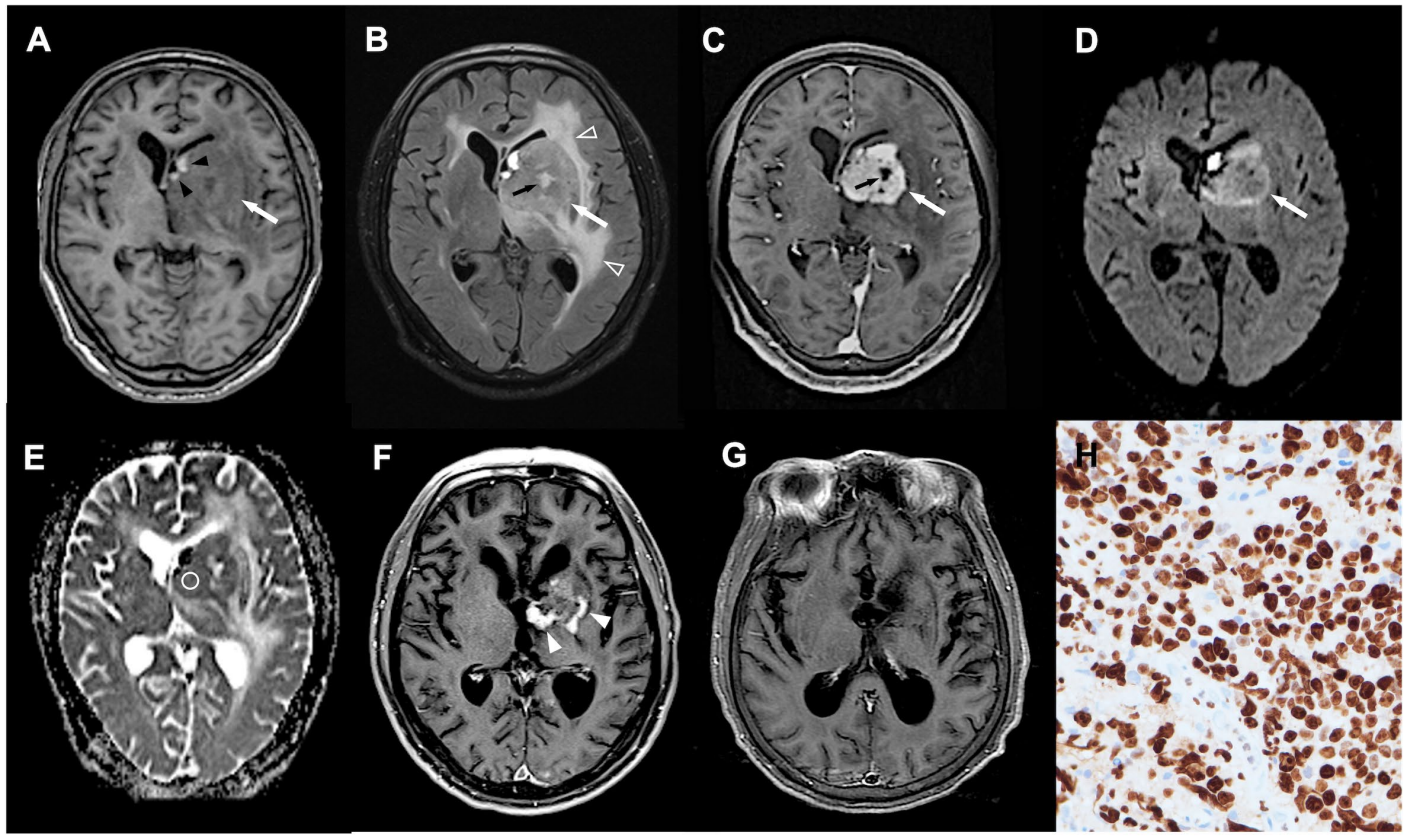
A 70-year-old man with pathologically confirmed PCNSL. **(A–C)** Axial T1WI **(A)**, T2-fluid attenuated inversion recovery (FLAIR) **(B)**, and CE T1WI **(C)** showing an enhanced tumor (white arrow) arising from left basal ganglia, with spot intratumoral hemorrhage (black arrowhead), peritumoral edema (open arrowhead), and focal intratumoral necrosis (black arrow). **(D)** DWI showing hyperintensity in the tumor mass (white arrow). **(E)** Measured ADC value (circular region of interest) was 0.75 × 10 mm^2^/s and ADC ratio was 1.19. **(F,G)** Obvious shrinkage of tumor mass (white arrowhead) with complete response **(G)** was observed after first-line chemotherapy. No tumor recurrence was detected at 13 months follow-up after treatments. **(H)** The proliferation rate determined from the immunohistochemical Ki-67 labeling index was 90% (original magnification 400).

**Figure 3:**
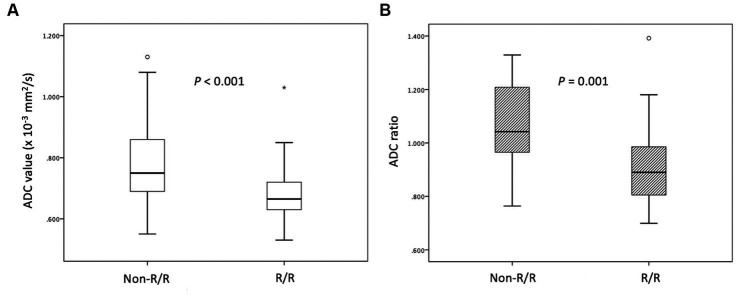
Box plots of **(A)** ADC values and **(B)** ADC ratios in PCNSL with and without relapsed/refractory (R/R) disease. Lower **(A)** ADC values and **(B)** ADC ratios in the R/R group (median ADC value, 0.66 × 10^−3^ mm^2^/s; median ADC ratio, 0.88) compared with the non-P/R group (median ADC value, 0.75 × 10^−3^ mm^2^/s; median ADC ratio, 1.04) (Mann–Whitney U test, *p* < 0.05) were observed. Boxes indicate the interquartile range, and whiskers indicate the range. The horizontal line represents the median in each box. Circles represent outliers, defined as distances >1.5 times the interquartile range less than the first quartile or greater than the third quartile. The star represents an extreme value, defined as a distance greater than 3 times the interquartile range above the third quartile.

The clinical data and MRI features of the PCNSL with and without R/R are summarized in Table 1. Of the 52 patients, 31 (31/52, 59.6%) patients achieved CR, 12 (12/52, 23.1%) achieved PR, 2 (2/52, 3.8%) remained SD, and 7 (7/52, 13.5%) showed PD after first-line chemotherapy. The objective response rate was 82.7% (43/52). A total of 24 (24/52, 46.2%) PCNSL patients developed R/R after treatments (Figures 1, 2). Female sex and non-CR after first-line chemotherapy were more frequent in the R/R group than in those without R/R (*p* < 0.05). The median ADC values and ADC ratios were lower in the R/R group than in those without R/R (*p* < 0.05) (Figure 3). The median follow-up duration for all patients was 26.3 months. In the 24 patients with R/R, the median time to R/R was 13 months. Eighteen (18/52, 34.6%) patients died, and the median time to death was 20.9 months.

**Table 1:** The clinical data and MRI findings of patients with and without relapsed/refractory (R/R) primary central nervous system lymphoma (PCNSL).

	R/R	Non-R/R	*p*-value
Number of patients	24	28	
Sex			0.043*
Male	7 (29.2%)	16 (57.1%)	
Female	17 (70.8%)	12 (42.9%)	
Age (y)	62 (54, 70)	66 (56, 76)	0.419
Response to first-line HD-MTX (8 g/m2) based chemotherapy			0.002*
Complete response (CR)	9 (37.5%)	22 (78.6%)	
Partial response (PR)/Stable disease (SD)	8 (33.3%)	6 (21.4%)	
Progressive disease (PD)	7 (29.2%)	0	
Tumor location			0.794
Cerebral cortex	14 (58.3%)	18 (64.3%)	
Basal ganglia/thalamus/corpus callosum	8 (33.3%)	7 (25%)	
Cerebellum	2 (8.3%)	3 (10.7%)	
Ocular involvement	3 (12.5%)	2 (7.1%)	0.652
Enhancement			0.438
Homogeneous	12 (50%)	11 (39.3%)	
Heterogeneous	12 (50%)	17 (60.7%)	
Necrosis	9 (37.5%)	12 (42.9%)	0.695
Hemorrhagic change	7 (29.2%)	8 (28.6%)	0.962
Peritumoral edema	22 (91.7%)	26 (92.9%)	1
Leptomeningeal seeding	4 (16.7%)	2 (7.1%)	0.397
Multiple lesions	12 (50%)	12 (42.9%)	0.606
Maximal tumor diameter (cm)	3.85 (3.15, 4.55)	3.30 (2.30, 4.30)	0.406
High DWI signal	21 (87.5%)	23 (82.1%)	0.711
ADC value (×10^−3^ mm^2^/s)	0.66 (0.605, 0.705)	0.75 (0.665, 0.835)	<0.001*
ADC ratio	0.88 (0.793, 0.975)	1.04 (0.915, 1.170)	0.001*
LDH (units/L)	208 (170, 247)	226 (145, 306)	0.391
Ki-67 (%)	80 (70, 90)	87.5 (79.5, 95.5)	0.779
**Recurrence site**			
CNS	20 (83.3%)		
Isolated systemic	2 (8.3%)		
Both CNS and systemic	2 (8.3%)		
Death	11 (45.8%)	7 (25%)	0.115
Follow-up time (months)	30.4 (17.7, 43)	21.2 (6.4, 37.9)	0.186

Continuous variables were presented as median and interquartile range (IQR).

* Statistical difference (*p* < 0.05).

### ROC curve analyses of ADC

**Figure 4:**
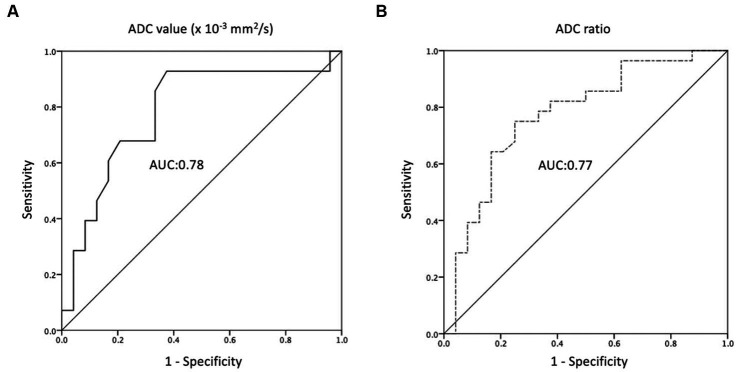
Receiver operating characteristic (ROC) curves of **(A)** ADC value and **(B)** ADC ratio for the prediction of R/R PCNSL, with optimal cut-off value of 0.68 × 10^−3^ mm^2^/s and 0.97, respectively. The AUCs of ADC value and ADC ratio in the prediction of P/R are 0.78 and 0.77, respectively.

The ROC analyses of the ADC values and ratios for differentiation between the R/R and non-R/R groups are shown in Figure 4. The cut-off points for the ADC value and ADC ratio were 0.68 × 10^−3^ mm^2^/s and 0.97, respectively. An AUC of 0.78 and 0.77 were obtained for the ADC value and ADC ratio, respectively.

### Recurrence rate and Cox proportional hazards regression analysis

**Figure 5:**
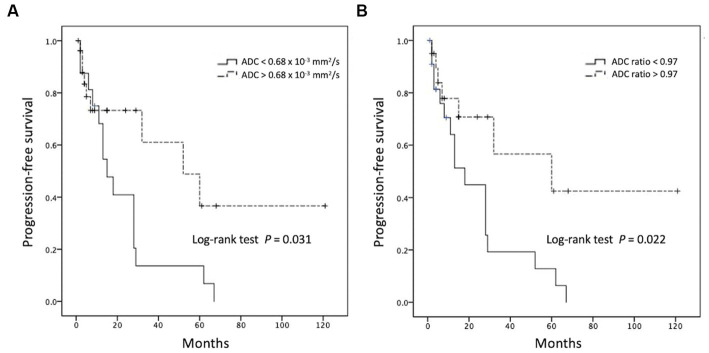
Kaplan–Meier curves showing overall trends of progression-free survival using cutoff points of **(A)** ADC value and **(B)** ADC ratio.

When comparing the tumor progression trends, patients with lower ADC values (less than cut-off value of 0.68 × 10^−3^ mm^2^/s) and lower ADC ratios (less than cut-off value of 0.97) exhibited shorter PFS (*p* < 0.05; Figure 5). The results of the Cox proportional hazards analyses are summarized in Table 2. Univariate analysis revealed statistically significant differences (*p* < 0.05) in sex, CR to first-line chemotherapy, and ADC value/ratio between the two groups. Furthermore, multivariate analysis showed that failure of CR to first-line chemotherapy and low ADC value were significant predictors of R/R (*p* < 0.05) with hazard ratios of 5.22 and 14.45, respectively. Additional data of univariate and multivariate Cox proportional hazard analyses for R/R and overall survival (OS) are supplied in Supplementary files S2, S3.

**Table 2:** Cox proportional hazards analysis for R/R PCNSL.

	Univariate analysis		Multivariate analysis	
	HR (95% CI) for R/R PCNSL	*p*-value	HR (95% CI) for R/R PCNSL	*p*-value
Sex (fraction female)	3.24 (1.02, 10.28)	0.046*	2.74 (0.61, 12.28)	0.189
Age (years)	0.99 (0.95, 1.04)	0.725		
Failure of CR to first-line chemotherapy	6.11 (1.80, 20.78)	0.004*	5.22 (1.12, 24.33)	0.035*
Tumor location (cerebral cortex)	0.78 (0.25, 2.39)	0.660		
Ocular involvement	1.86 (0.28, 12.16)	0.519		
Heterogeneous enhancement	0.64 (0.23, 1.80)	0.395		
Necrosis	0.80 (0.26, 2.44)	0.695		
Hemorrhagic change	1.03 (0.31, 3.43)	0.962		
Peritumoral edema	0.85 (0.11, 6.51)	0.873		
Leptomeningeal seeding	2.60 (0.43, 15.65)	0.297		
Multiple lesions	1.33 (0.45, 3.99)	0.607		
Maximal tumor diameter (cm)	1.02 (0.72, 1.45)	0.896		
High DWI signal	1.52 (0.32, 7.16)	0.595		
ADC < 0.68 × 10^−3^ mm^2^/s (cut-off value)	21.67 (4.13, 113.81)	<0.001*	14.45 (2.47, 84.41)	0.003*
ADC ratio < 0.97 (cut-off value)	7.29 (2.14, 24.86)	0.002*		
Ki-67 (%)	0.99 (0.97, 1.03)	0.808		
LDH (units/L)	0.99 (0.99, 1.00)	0.212		

* Statistical difference (*p* < 0.05).

## Discussion

The present study analyzed the pretreatment clinical and MRI features to predict clinical outcomes in PCNSL, emphasizing ADC values in DWI, and the results showed that CR after first-line therapy and low pretreatment ADC values/ratios were significantly associated with R/R PCNSL. Although a few small studies had previously reported the application of ADC for evaluation of clinical outcomes in PCNSL (14–16), the present results are the first to offer pretreatment ADC threshold values for the prediction of R/R in PCNSL.

PCNSL is a highly aggressive NHL, and the incidence has increased in individuals older than 60 years (25). Although some PCNSL responds favorably to chemotherapy and radiotherapy, the optimal treatment strategy for PCNSL remains controversial (4, 7). On conventional MRI, PCNSL typically appears as an enhancing mass lesion with a supratentorial location, and it preferentially involves the deep cerebral parenchyma such as basal ganglia (26). The present study showed similar MRI features for PCNSL. So far, rare well-established imaging biomarkers have been predictive of clinical outcomes in PCNSL. Traditionally, an assessment of solid tumor therapeutic effects depends on comparison of tumor size changes by CT or MR imaging obtained before and after the treatments. However, tumor size change is insensitive to early treatment changes and cannot monitor such effects at the cellular level of tumor tissue (27).

DWI has been widely used as a cancer imaging biomarker to evaluate various intracranial and extracranial tumors as well as to predict their grade of differentiation (12). It provides biomedical information based on measurement of thermally induced random Brownian motion of water molecules within a voxel of tissue as shown by ADC values (13). Therefore, unusual findings on ADC values may be an early predictor of biological abnormality (12). ADC values decrease in tissue with restricted diffusion, such as highly cellular tumors; thus, low ADC values are associated with poorer clinical outcomes in various cancers (12). The combination of conventional MR imaging with quantitative ADC values enables the assessment of morphologic and physiologic changes during the same examination. The extremely high DWI signal is a characteristic MRI feature in PCNSL due to the high cell density (28). Previous studies have shown a significant inverse correlation between cellularity and ADC values in PCNSL, suggesting that ADC is a surrogate marker of tumor proliferation (14, 29). Although ADC has shown promising results in the evaluation of response to therapy for non-Hodgkin lymphoma (15), the validity of pretherapeutic ADC as a predictor of clinical outcomes in patients with PCNSL remains controversial. Barajas et al. (14) first reported that lower ADC_25%_ (lower than median value of 0.69 × 10^−3^ mm^2^/s) and ADC_min_ (lower than median value of 0.38 × 10^−3^ mm^2^/s) were associated with shorter PFS and overall survival (OS) in 18 PCNSL patients, and an inverse correlation was found between ADC value and tumor cellular density. Further, Wieduwilt et al. (30) and Valles et al. (31) used the same cut-off points (14) to reinforce the validity of ADC_min_ as a prognostic biomarker in 23 and 25 PCNSL patients, respectively. Recently, Baek et al. (16) reported that low ADC was an independent unfavorable prognostic factor in 52 PCNSL patients, but no definitive threshold value was mentioned in ADC. In contrast, in a multicenter study by Morris et al. (17), no differences were found in baseline ADC values for predicting therapeutic response, PFS, and OS in 52 PCNSL cases. Huang et al. (15) also reported no significant differences in pretreatment ADC_min_ between the PD, CR, and PR groups in 35 PCNSL patients with methotrexate-based chemotherapy. Instead, ADC_min_ changes after early therapy may more precisely predict treatment response (15). Most of the above-mentioned studies included relatively few cases and still rare available pretreatment ADC data could be applied to predict R/R PCNSL.

The present study revealed that 46.2% (24/52) patients had R/R PCNSL after treatments, which was similar to that reported in a previous study (32). In addition, low ADC value is also a significant predictor for poor OS in the present study. Baek et al. (16) reported 69.7% in 3-year OS rate in 52 PCNSL patients, and similar results was observed in the present study. Unlike high-grade glioma, gross total surgical resection of enhanced tumors shows no significant improvement in clinical outcomes for PCNSL (33). Although increased intratumoral expression of activated *STAT6* based on histopathologic analysis is associated with poor clinical outcomes in PCNSL (34), quantification of *STAT6* expression requires an invasive procedure and is not widely available clinically. The ability to predict prognosis in PCNSL based on noninvasive imaging biomarkers may have a significant impact on clinical management. Traditionally, the response to MTX-based chemotherapy is monitored by morphological changes in tumor size on CE MRI. However, clinical signs of R/R PCNSL can occur earlier than the presentation of change in tumor size due to molecular and cellular changes (19, 20). For PCNSL patients with high risks of R/R after first-line MTX-based chemotherapy, changes in chemotherapy agents and further consolidation therapy with auto-SCT or WBRT should be considered in treatment decisions. Therefore, the pretherapeutic ADC measurement offers an opportunity to modify the initial treatment regimen to improve the clinical outcome and decrease the morbidity associated with prolonged and ineffective treatments. Some authors have also reported serial ADC measurements during MRI follow up, which offers useful information for assessing early therapeutic response in PCNSL (14, 15). On the other hand, precise localization of enhancing tumors with relatively low ADC values may minimize the rate of false-negative biopsy since low ADC values are associated with high tumor cellularity in PCNSL (14). Therefore, pretreatment ADC data has the potential to offer additional values in both tumor tissue sampling and prediction of R/R in PCNSL. The technique for measuring ADC values is easy and available in most hospitals without needing to use contrast medium.

To the best of our knowledge, this is the first study analyzing the pretreatment ADC threshold values for predicting R/R in PCNLS. However, the present study still has several limitations. The retrospective nature of the present study may result in selection bias. As in other ROI-based studies, subjective placement of the ROIs may have influenced the accuracy of the ADC measurements. Although similar parameters were used in each MRI machine, the ADC values may have differed among the various MRI scanners. The correlation of ADC and tumor cellularity was not performed in the present study. Finally, genetic profiling should be conducted in the future to clarify associations between molecular subgroups and PCNSL prognosis.

## Conclusion

The present study showed that patients’ response to first-line chemotherapy and pretreatment ADC values are significant predictors of clinical outcomes in PCNSL. The pretherapeutic quantitative ADC values and ratios offer valuable information for treatment planning in PCNSL, including the choice of chemotherapy agents, and decisions on further consolidation therapy with stem cell transplantation or radiotherapy.

## Data availability statement

The raw data supporting the conclusions of this article will be made available by the authors, without undue reservation.

## Ethics statement

The studies involving human participants were reviewed and approved by Institutional Review Board of the Chi Mei Medical Center (IRB no.: 10902-009). Written informed consent for participation was not required for this study in accordance with the national legislation and the institutional requirements.

## Author contributions

C-CK: conceptualization and methodology. H-CC, S-WL, C-YC, and Y-CL: data curation. H-CC and C-CK: formal analysis and validation. H-CC: resources and writing—original draft. K-CH: software. L-RY: supervision. C-CK and J-HC: writing—review and editing. All authors contributed to the article and approved the submitted version.

## Funding

This work was supported by the Chi Mei Medical Center, Tainan, Taiwan (no. CMFHR 112015). The funders had no role in study design, data collection and analysis, decision to publish, or preparation of the manuscript.

## Conflict of interest

The authors declare that the research was conducted in the absence of any commercial or financial relationships that could be construed as a potential conflict of interest.

## Publisher’s note

All claims expressed in this article are solely those of the authors and do not necessarily represent those of their affiliated organizations, or those of the publisher, the editors and the reviewers. Any product that may be evaluated in this article, or claim that may be made by its manufacturer, is not guaranteed or endorsed by the publisher.
