# Supplementary material for: Pretreatment diffusion-weighted imaging for prediction of relapsed and refractory primary central nervous system lymphoma
Source: Front Neurol. 2023 Aug 10;14:1227607. doi: 10.3389/fneur.2023.1227607 (PMC10447899; doi:10.3389/fneur.2023.1227607)
Supplement: Supplementary file 1 [file Data_Sheet_1.docx]

Supplementary Material

Pretreatment Diffusion-Weighted Imaging for Prediction of Relapsed and Refractory Primary Central Nervous System Lymphoma

Hsi-Cheng Chien^1†^, Lee-Ren Yeh^2,3,4†^, Kuo-Chuan Hung^5,6^, Sher-Wei Lim^7,8^, Chung-Yu Cheng^2^, Yu-Chang Lee^2^, Jeon-Hor Chen^2,9^, Ching-Chung Ko^1,10*^

^†^ Hsi-Cheng Chien and Lee-Ren Yeh equally contributed to the study.

*** Correspondence:**

Ching-Chung Ko: kocc0729@gmail.com

# Supplementary file 1

**MRI Protocols**

The protocols of 1.5T (Siemens, MAGNETOM Avanto) MR imaging were as the following: axial T1-weighted spin-echo (T1WI)(TR/TE, 2000/7 ms; FOV, 22 cm; slice thickness/spacing, 5 mm/6.5 mm; matrix, 320 x 224; voxel size, 0.69 x 0.98 x 5 mm^3^), T2-weighted imaging (T2WI) (fast spin-echo)(3730/108 ms; FOV, 22 cm; slice thickness/spacing, 5 mm/6.5 mm; matrix, 384 x 261; voxel size, 0.57 x 0.84 x 5 mm^3^), fluid attenuated inversion recovery (FLAIR) (9000/92 ms; FOV, 22 cm; slice thickness/spacing, 5 mm/6.5 mm; matrix, 256 x 209; voxel size, 0.86 x 1.05 x 5 mm^3^), and T2-weighted gradient-recalled echo (GRE) (830/26 ms; FOV, 22 cm; slice thickness/spacing, 5 mm/6.5 mm; matrix, 256 x 157; voxel size, 0.86 x 1.4 x 5 mm^3^). Contrast-enhanced images obtained in axial and coronal T1WI with fat saturation (2000/8 ms; FOV, 22 cm; slice thickness/spacing, 5 mm/6.5 mm; matrix, 320 x 256; voxel size, 0.69 x 0.86 x 5 mm^3^) were performed after intravenous administration of 0.1 mmol/kg of body weight of gadobutrol (Gadovist; Schering, Berlin, Germany) or gadoterate meglumine (Dotarem; Guerbet, Villepinte, France). The DWI was performed by applying sequentially in the x, y, and z directions with the following parameters: TR/TE, 3800/105 ms; FOV, 23cm; flip angle, 90 degrees, slice thickness/spacing, 5 mm/6.5 mm; matrix, 192 x 192; voxel size, 1.2 x 1.2 x 5 mm^3^; b = 0, 500, and 1000 sec/mm^2^. ADC maps were obtained from these imaging data.

The protocols of 1.5T (Siemens, MAGNETOM Aera) MR imaging were as the following: axial T1-weighted spin-echo (T1WI)(TR/TE, 2000/9 ms; FOV, 22 cm; slice thickness/spacing, 5 mm/6.5 mm; matrix, 320 x 224; voxel size, 0.69 x 0.98 x 5 mm^3^), T2-weighted imaging (T2WI) (fast spin-echo)(3760/99 ms; FOV, 22 cm; slice thickness/spacing, 5 mm/6.5 mm; matrix, 448 x 284; voxel size, 0.49 x 0.77 x 5 mm^3^), fluid attenuated inversion recovery (FLAIR) (9000/86 ms; FOV, 22 cm; slice thickness/spacing, 5 mm/6.5 mm; matrix, 320 x 218; voxel size, 0.69 x 1.01 x 5 mm^3^), and T2-weighted gradient-recalled echo (GRE) (830/26 ms; FOV, 22 cm; slice thickness/spacing, 5 mm/6.5 mm; matrix, 256 x 157; voxel size, 0.86 x 1.4 x 5 mm^3^). Contrast-enhanced images obtained in axial and coronal T1WI with fat saturation (2160/9 ms; FOV, 22 cm; slice thickness/spacing, 5 mm/6.5 mm; matrix, 320 x 224; voxel size, 0.69 x 0.98 x 5 mm^3^) were performed after intravenous administration of 0.1 mmol/kg of body weight of gadobutrol (Gadovist; Schering, Berlin, Germany) or gadoterate meglumine (Dotarem; Guerbet, Villepinte, France). The DWI was performed by applying sequentially in the x, y, and z directions with the following parameters: TR/TE, 5020/69 ms; FOV, 23cm; flip angle, 90 degrees, slice thickness/spacing, 5 mm/6.5 mm; matrix, 192 x 192; voxel size, 1.2 x 1.2 x 5 mm^3^; b = 0, 500, and 1000 sec/mm^2^. ADC maps were obtained from these imaging data.

The protocols of 1.5T (GE Healthcare, Signa) MR imaging were as following: axial T1WI (TR/TE, 2115/10 ms; FOV, 22 cm; slice thickness/spacing, 5 mm/6.5 mm; matrix, 288 x 224; voxel size, 0.76 x 0.98 x 5 mm^3^), T2WI (3417/109 ms; FOV, 22 cm; slice thickness/spacing, 5 mm/6.5 mm; matrix, 320 x 256; voxel size, 0.69 x 0.86 x 5 mm^3^), FLAIR (9002/140 ms; FOV, 22 cm; slice thickness/spacing, 5 mm/6.5 mm; matrix, 256 x 192; voxel size, 0.86 x 01.15 x 5 mm^3^), and T2-weighted GRE (450/15 ms; FOV, 24 cm; slice thickness/ spacing, 5 mm/6.5 mm; matrix, 288 x 192; voxel size, 0.83 x 1.25 x 5 mm^3^). Contrast-enhanced axial and coronal T1WI with fat saturation (2115/8 ms; FOV, 22 cm; slice thickness/spacing, 5 mm/6.5 mm; matrix, 256 x 224; voxel size, 0.86 x 0.98 x 5 mm^3^) with intravenous administration of 0.1 mmol/kg of Gadovist or Dotarem. The DWI was performed by applying sequentially in the x, y, and z directions with the following parameters: TR/TE, 6600/73 ms; flip angle, 90 degrees, slice thickness/spacing, 5 mm/6.5 mm; b = 0 and 1000 sec/mm^2^. ADC maps were obtained from these imaging data. TR/TE, 6600/72 ms; FOV, 25cm; flip angle, 90 degrees, slice thickness/spacing, 5 mm/6.5 mm; matrix, 128 x 256; voxel size, 1.95 x 0.98 x 5 mm^3^; b = 0 and 1000 sec/mm^2^. ADC maps were obtained from these imaging data.

The protocols of 3T (GE Healthcare, Discovery MR750) MR imaging were as following: axial T1WI (TR/TE, 3400/24 ms; FOV, 22 cm; slice thickness/spacing, 5 mm/6.5 mm; matrix, 352 x 224; voxel size, 0.63 x 0.98 x 5 mm^3^), T2WI (5200/102 ms; FOV, 22 cm; slice thickness/spacing, 5 mm/6.5 mm; matrix, 384 x 320; voxel size, 0.57 x 0.69 x 5 mm^3^), FLAIR (10000/95 ms; FOV, 22 cm; slice thickness/spacing, 5 mm/6.5 mm; matrix, 320 x 192; voxel size, 0.69 x 1.15 x 5 mm^3^), and T2-weighted GRE (567/20 ms; FOV, 22 cm; slice thickness/ spacing, 5 mm/6.5 mm; matrix, 256 x 160; voxel size, 0.86 x 1.38 x 5 mm^3^). Contrast-enhanced axial and coronal T1WI with fat saturation (2215/ 22 ms; FOV, 22 cm; slice thickness/spacing, 5 mm/6.5 mm; matrix, 320 x 224; voxel size, 0.69 x 0.98 x 5 mm^3^) after intravenous administration of 0.1 mmol/kg of Gadovist or Dotarem. The DWI was performed by applying sequentially in the x, y, and z directions with the following parameters: TR/TE, 8000/64 ms; FOV, 22 cm; flip angle, 90 degrees, slice thickness/spacing, 5 mm/6.5 mm; matrix, 128 x 160; voxel size, 1.72 x 1.38 x 5 mm^3^; b = 0, 1000, and 1500 sec/mm^2^. ADC maps were obtained from these imaging data.

**Supplementary file 2**

**Cox proportional hazards analysis for R/R PCNSL**

|  | Univariate Analysis | | Multivariate Analysis | |  | Multivariate Analysis ^a^ | |
| --- | --- | --- | --- | --- | --- | --- | --- |
|  | **HR (95 % CI) for R/R PCNSL** | ***p* value** | **HR (95 % CI) for R/R PCNSL** | ***p* value** | | **HR (95 % CI) for R/R PCNSL** | ***p* value** |
| Sex (fraction female) | 3.24 (1.02, 10.28) | 0.046* | 7.18 (0.71, 72.53) | | 0.095 | 2.74 (0.61, 12.28) | 0.189 |
| Age (years) | 0.99 (0.95, 1.04) | 0.725 | 1.00 (0.94, 1.06) | | 0.974 |  |  |
| Failure of CR to first-line chemotherapy | 6.11 (1.80, 20.78) | 0.004* | 0.20 (0.04, 0.95) | | 0.043* | 5.22 (1.12, 24.33) | 0.035* |
| Tumor location (cerebral cortex) | 0.78 (0.25, 2.39) | 0.660 | 0.85 (0.18, 4.10) | | 0.838 |  |  |
| Ocular involvement | 1.86 (0.28, 12.16) | 0.519 | 0.74 (0.06, 9.76) | | 0.821 |  |  |
| Heterogeneous enhancement | 0.64 (0.23, 1.80) | 0.395 | 0.42 (0.09, 1.96) | | 0.270 |  |  |
| Necrosis | 0.80 (0.26, 2.44) | 0.695 | 0.55 (0.11, 2.80) | | 0.468 |  |  |
| Hemorrhagic change | 1.03 (0.31, 3.43) | 0.962 | 0.83 (0.16, 4.34) | | 0.826 |  |  |
| Peritumoral edema | 0.85 (0.11, 6.51) | 0.873 | 0.50 (0.05, 4.88) | | 0.548 |  |  |
| Leptomeningeal seeding | 2.60 (0.43, 15.65) | 0.297 | 1.28 (0.14, 11.71) | | 0.829 |  |  |
| Multiple lesions | 1.33 (0.45, 3.99) | 0.607 | 0.58 (0.12, 2.84) | | 0.504 |  |  |
| Maximal tumor diameter (cm) | 1.02 (0.72, 1.45) | 0.896 | 0.79 (0.47, 1.34) | | 0.386 |  |  |
| High DWI signal | 1.52 (0.32, 7.16) | 0.595 | 0.92 (0.13, 6.52) | | 0.931 |  |  |
| ADC < 0.68 x 10^-3^ mm^2^/s | 21.67 (4.13, 113.81) | < 0.001* | 11.69 (1.50, 90.93) | | 0.019* | 14.45 (2.47, 84.41) | 0.003* |
| ADC ratio < 0.97 | 7.29 (2.14, 24.86) | 0.002* | 0.71 (0.13, 3.97) | | 0.694 |  |  |
| Ki-67 (%) | 0.99 (0.97, 1.03) | 0.808 | 1.01 (0.97, 1.05) | | 0.676 |  |  |
| LDH (units/L) | 0.99 (0.99, 1.00) | 0.212 | 0.99 (0.99, 1.00) | | 0.029* |  |  |

* Statistical difference (*p* < 0.05).

**^a^** Only variables with a *p* value < 0.05 in univariate analysis were carried forward to the multivariate analysis.

**Supplementary file 3**

**Cox proportional hazards analysis for overall survival (OS) in PCNSL**

|  | Univariate Analysis | | Multivariate Analysis | |
| --- | --- | --- | --- | --- |
|  | **HR (95 % CI) for OS** | ***p* value** | **HR (95 % CI) for OS** | ***p* value** |
| Sex (fraction female) | 0.72 (0.22, 2.29) | 0.573 |  |  |
| Age (years) | 0.99 (0.95, 1.04) | 0.720 |  |  |
| Failure of CR to first-line chemotherapy | 0.27 (0.08, 0.88) | 0.030* | 0. 34 (0.09, 1.23) | 0.100 |
| Tumor location (cerebral cortex) | 0.33 (0.10, 1.09) | 0.070 |  |  |
| Ocular involvement | 9.43 (0.97, 92.06) | 0.054 |  |  |
| Heterogeneous enhancement | 0.99 (0.31, 3.12) | 0.982 |  |  |
| Necrosis | 0.91 (0.28, 2.92) | 0.873 |  |  |
| Hemorrhagic change | 0.59 (0.16, 2.23) | 0.439 |  |  |
| Peritumoral edema | 1.69 (0.16, 17.71) | 0.662 |  |  |
| Leptomeningeal seeding | 1.00 (0.14, 7.10) | 1.000 |  |  |
| Multiple lesions | 0.90 (0.26, 3.14) | 0.869 |  |  |
| Maximal tumor diameter (cm) | 0.94 (0.65, 1.36) | 0.739 |  |  |
| High DWI signal | 0.86 (0.18, 4.11) | 0.852 |  |  |
| ADC value < 0.65 x 10^-3^ (mm^2^/s) | 8.27 (1.83, 37.28) | 0.006* | 6.81 (1.44, 32.16) | 0.016* |
| ADC ratio < 0.90 | 4.06 (1.20, 13.78) | 0.025* |  |  |
| Ki-67 (%) | 1.01 (0.98, 1.04) | 0.716 |  |  |
| LDH (units/L) | 1.00 (1.00, 1.01) | 0.322 |  |  |

*Statistical difference (*p* < 0.05).
